# High salt tolerance but no local adaption to road salts in *Tetrahymena* ciliates

**DOI:** 10.17912/micropub.biology.001862

**Published:** 2026-01-15

**Authors:** Zadia Swain, Marc Aboulehaf, Karissa Plum, Rebecca A. Zufall

**Affiliations:** 1 Department of Biology and Biochemistry, University of Houston, Houston, Texas, United States

## Abstract

Road salt application improves road safety but leads to salinization of nearby freshwater ecosystems. If populations readily adapt to their local salinity environment, then we expect to find differences in salt tolerance between populations found near vs. far from roads. We determined the salt tolerance of 10 wild strains of the freshwater microbial eukaryote *Tetrahymena* . We found no significant correlation between salt tolerance and road distance, suggesting that these populations are not locally adapted to their salinity environment. This result may be due to the unexpectedly high salt tolerance across lines, unknown patterns of migration between ponds, or sampling conditions.

**Figure 1. Survival of Tetrahymena strains at varying salt concentrations does not correlate with distance from nearest road:**
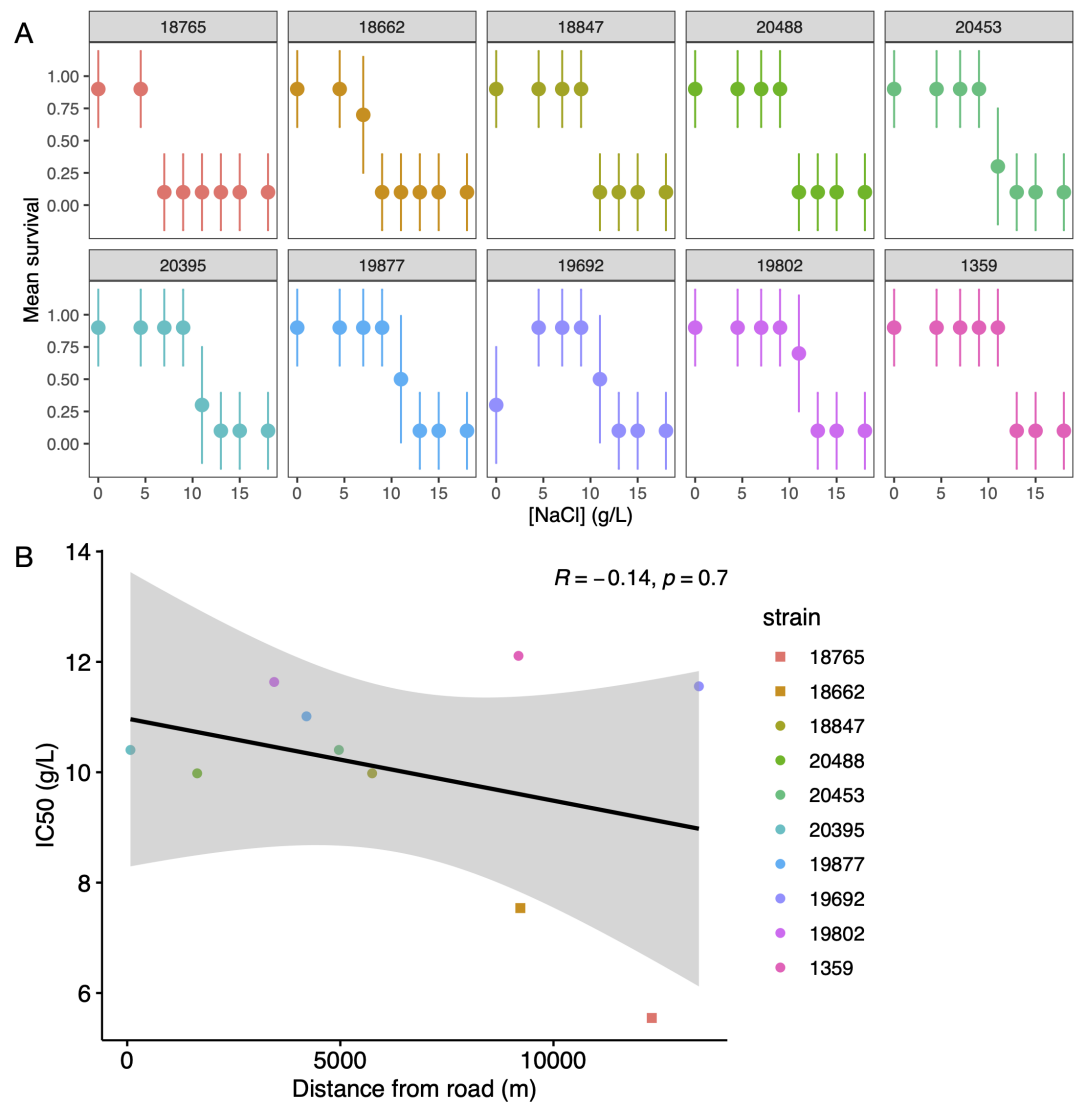
A. Mean survival and 95% confidence intervals of each strain measured at 0, 4.5, 7, 9, 11, 13, 15, and 18 g/L NaCl. Strains are ordered by increasing salt tolerance. B. Correlation between IC50 and distance from nearest road. Linear regression, 95% confidence interval, and Spearman correlation coefficient are shown. *T. ellioti* are represented with squares; *T. thermophila* with circles.

## Description

Road deicing salts are commonly used in parts of the world that experience cold, snowy winters. Road salts improve driving safety, but since the start of their use in the 1940s, salinization of freshwater ecosystems near roadways has been dramatically increasing (Corsi et al., 2010; Dugan and Arnott, 2023; Hintz et al., 2022; Kaushal et al., 2005). Increased salinity in freshwater ecosystems can lead to acute and chronic toxicity, changes in population dynamics, and reduced biodiversity (Corsi et al., 2010; Kaushal et al., 2005; Searle et al., 2016; Szklarek et al., 2022).

Previous studies using experimental evolution have demonstrated that the freshwater species *Daphnia pulex* and *Tetrahymena thermophila* have the capacity to rapidly evolve increased tolerance to road salts (Coldsnow et al., 2017; Hintz et al., 2018; Zufall et al., 2025). These results suggest that natural populations may be undergoing similar changes in salt tolerance in response to road salt application. Indeed, a study in salamanders found evidence of local adaptation, possibly due to elevated salinity, in road-adjacent populations (Brady, 2012). However, the extent to which road salt application results in local adaptation of populations near or distant from salted roads remains unknown.

*Tetrahymena* are a genus of ciliates, microbial eukaryotes, that inhabit freshwater ecosystems worldwide and serve as an important link in the microbial food loop (Doerder, 2019; Fenchel, 2008; Lynn, 2012; Weisse, 2017). *T. thermophila* , the primary focus of this study, is endemic to ponds and streams in the northeastern United States (Zufall et al., 2013), where road salt is commonly applied during the winter. To test whether *Tetrahymena* are locally adapted to their salinity environments, we measured the salt tolerance of 10 isolates of *Tetrahymena* (8 *T. thermophila* and 2 *T. ellioti* ) that had been collected at varying distances from the nearest road.

Salt tolerance was measured by survival probability in a range of concentrations of NaCl, the most commonly used road salt. Most isolates survived well in salt-free and low-salt conditions, with survivorship dropping off rapidly at higher concentrations ( Fig. 1A ). Interestingly, strain 19692 is able to survive at some of the highest salt concentrations, but experienced decreased survival in the salt-free environment. This suggests a trade-off between survival in high-salt and no-salt conditions, similar to what was seen in experimentally evolved populations of this species (Zufall et al., 2025).

Estimates of half-maximal inhibitory concentrations of NaCl (IC50) demonstrate significant differences among lines ( Fig. 1B ). The two isolates with the lowest IC50 are both *T. ellioti* , suggesting that *T. thermophila* tend to have higher salt tolerance than *T. ellioti* . The range of IC50 among *T. thermophila* (10.0 – 12.1 g/L) is nearly half the concentration of seawater (~35 g/L). This is much higher than expected for a freshwater species, suggesting that this species may be broadly adapted to a high salinity environment. Interestingly, the source of commonly used laboratory strains of *T. thermophila* , was originally collected from a tidal pond in Woods Hole, MA (Doerder and Brunk, 2012) indicating that they can survive in salty habitats.

Finally, the distance from nearest road was estimated for each strain and tested for a correlation with IC50 ( Fig. 1B ). If there was local adaptation, we would expect to see a negative correlation between IC50 and distance from road. While the measured correlation is negative, it is not significant (Spearman rank correlation; R = -0.14, p = 0.71). In addition, if we remove the two strains of *T. ellioti* the correlation becomes positive, though still nonsignificant (Spearman rank correlation; R = 0.38, p = 0.36).

Together these results suggest that *Tetrahymena* are not locally adapted to salinity based on nearness to salted roads. Several factors are likely to contribute to this result. First, all of our *T. thermophila* isolates have very high salt tolerance. The highest salinities in freshwater systems in the northeastern United States have been reported to range from about 1-8 g/L NaCl (Brady and Benoit, 2025; Huber et al., 2024; Kaushal et al., 2005), lower than all but the *T. ellioti* IC50. Either *Tetrahymena* have already adapted to high salinities, or they have high salt tolerance as a byproduct of some other trait (e.g. Tarkington and Zufall, 2024). Second, the strains studied here have been collected over more than 20 years. While they were recovered from liquid nitrogen storage for this experiment, these strains were likely grown for many generations in the lab prior to storage. Zufall et al. (2025) showed that culturing under laboratory conditions can result in the evolution of increased salt tolerance, so our measured salt tolerance may not accurately reflect the tolerance of the strains when they were originally collected. In addition, level of salting on the nearby roads is likely to have changed over the past 20 years. Recently collected samples are needed to test whether changes in road salting patterns and/or lab culture has affected the salt tolerance of these strains. Finally, while little is known about the migration patterns of *Tetrahymena* (but see Zufall et al., 2013), it seems likely that individuals can be transported between ponds (accidentally by humans or other animals) up to 5-10 km or more. Thus, the pond that a strain was sampled from may not represent the evolutionary history of salt exposure that we infer. Further studies of the natural and adaptive history of *Tetrahymena* are necessary to determine how these populations have, and will, respond to increasing salinity due to road salt application.

## Methods

*Study system and culture conditions*

All strains were obtained from the *Tetrahymena* Stock Center (see Reagents). Cells were grown in a standard nutrient-rich *Tetrahymena* medium, SSP, with added penicillin-streptomycin-amphotericin solution (Cassidy-Hanley, 2012; Gorovsky et al., 1975) for all assays, either with or without added salts as described below.

*Growth assays*

Isolates were grown at room temperature in salt-free SSP in 6-well plates for 3-7 days. Once cultures reached mid-log phase, 500 cells of each strain were placed in a randomized position on a 96-well plate. 8 replicates of each strain were placed into a given salt concentration in a final volume of 180 µL. Each 96-well plate was divided vertically in half, with a different salt concentration on each half. All strains were assayed for survival at 8 concentrations of NaCl: 0, 4.5, 7, 9, 11, 13, 15, and 18 g/L. To determine whether populations can survive and grow at each salt concentration, changes in cell density were determined by OD _650_ measurements on a microplate reader every 10 minutes, with shaking, for 6 days. A culture was considered to have survived if sufficient cell division occurred to result in detectable OD values after 6 days. Survival probability was based on the fraction of replicate cultures that survived (see *Data Analysis* ).

*Distance from road estimates*

Collection locations of each strain were obtained from the *Tetrahymena* Stock Center and mapped on Google Earth. The closest main road was identified via satellite imagery, based on its characteristics as a public path made of asphalt concrete and pavement markings. Distances were measured on the satellite map using the path feature, which allows users to draw a line between two points to calculate the distance between them.

*Data analysis*

Data were analyzed similar to Zufall et al. (2025). Briefly, microplate reader data were analyzed with the R package growthrates (Petzoldt, 2022) using the Huang (2011) growth model. Survival was determined based on whether a growth model fit the data with r ^2^ > 0.90. Wells with r ^2^ < 0.90 did not reach OD values above the detectable threshold, indicating little cell division and likely cell death, which was confirmed by visual inspection of wells. Survival probability was analyzed with a logistic model (survival ~ strain * NaCl concentration) using brglm_fit implementing mean bias reduction due to complete separation of the data (Kosmidis et al., 2020). Marginal means of survival were found using the R package modelbased (Makowski et al., 2025). The R package drc (Ritz et al., 2015) was then used to fit dose response curves (survival probability ~ NaCl concentration), extract IC50 values, and test for pairwise differences in IC50 values. The relationship between IC50 and distance from road was tested by Spearman correlation.

## Reagents

**Table:** 

**Strain**	**Species**	**Available from Tetrahymena Stock Center**
18765-1	*T. ellioti*	TSC_SD01676
18662-4	*T. ellioti*	TSC_SD01673
18847-3	*T. thermophila*	TSC_SD03079
20488-4	*T. thermophila*	TSC_SD01566
20453-1	*T. thermophila*	TSC_SD01561
20395-1	*T. thermophila*	TSC_SD01557
19877-1	*T. thermophila*	TSC_SD01555
19692-1	*T. thermophila*	TSC_SD03090
19802-1	*T. thermophila*	TSC_SD03093
1359-1	*T. thermophila*	TSC_SD03074
